# Suppressed Degradation Process of PBDB-TF-T1:BTP-4F-12-Based Organic Solar Cells with Solid Additive Atums Green

**DOI:** 10.1021/acsami.4c21699

**Published:** 2025-01-30

**Authors:** Zerui Li, Sergei Vagin, Jinsheng Zhang, Renjun Guo, Kun Sun, Xiongzhuo Jiang, Tianfu Guan, Matthias Schwartzkopf, Bernhard Rieger, Chang-Qi Ma, Peter Müller-Buschbaum

**Affiliations:** † Department of Physics, Chair for Functional Materials, TUM School of Natural Sciences, Technical University of Munich, James-Franck-Str. 1, 85748 Garching, Germany; ‡ i-Lab & Printable Electronics Research Center, Suzhou Institute of Nano-Tech and Nano-Bionics, Chinese Academy of Sciences (CAS), Ruoshui Road 398, SEID, SIP, Suzhou 215123, China; § Department of Chemistry, WACKER Chair of Macromolecular Chemistry, TUM School of Natural Sciences, Technical University of Munich, Lichtenbergstr. 4, 85748 Garching, Germany; ∥ Institute of Microstructure Technology, Karlsruhe Institute of Technology (KIT), Herrmann-von-Helmholtz-Platz 1, 76344 Karlsruhe, Germany; ⊥ Deutsches Elektronen-Synchrotron DESY, Notkestr. 85, 22607 Hamburg, Germany

**Keywords:** organic solar cell, green solvent, solid additive, *operando* study, GIWAXS and GISAXS

## Abstract

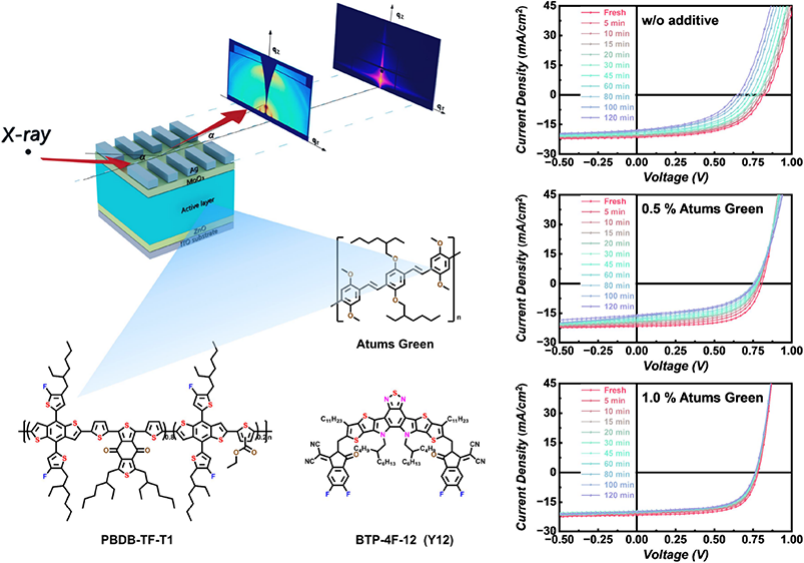

Solid additives have garnered significant attention due to their numerous advantages over liquid additives. This study explores the potential of the green-fluorescent conjugated polymer denoted Atums Green as a solid additive in green-solvent-based PBDB-TF-T1:BTP-4F-12 solar cells. Even tiny amounts of Atums Green doping significantly improve the device performance. For the reference solar cell without any additive, we find that device degradation is not caused by chemical redox reactions but by changes in crystallinity and microstructure evolution during aging in air under illumination. *Operando* GIWAXS and GISAXS are used to investigate the structure evolution. We discover a four-stage degradation process for the reference cell. In general, the lattice spacing and crystallite coherence length decrease, while the domain sizes increase, which causes the loss of shirt-circuit current *J*_SC_ and fill factor FF. Furthermore, a decomposition component is detected in GIWAXS and GISAXS, corresponding to the loss of the open-circuit voltage *V*_OC_. Atums Green doping effectively suppresses the evolution of crystallinity and domain sizes as well as the continuous decomposition, thereby enhancing the device stability under illumination in air. This finding reveals the kinetic degradation process of organic solar cells, establishes a correlation between the morphological properties and device performance, and further demonstrates the promising potential of Atums Green doping in organic solar cells.

## Introduction

Organic solar cells have shown various advantages, such as being lightweight, flexible, stretchable, and easy to fabricate, with champion efficiencies reaching over 20%.^1−3^ Forming a uniform separated nanomorphology of donor and acceptor phases in the active layer with proper dimensions is essential for the device performance.^4,5^ There are several strategies to optimize the film formation kinetics and morphology for achieving a better device performance and stability, such as changing solvents, utilizing additives and post-treatment (thermal annealing and solvent vapor annealing).^6−9^ Utilizing additives is one straightforward way to optimize the device performance and stability by inducing a miscibility regulation, thereby improving the film morphology, expanding the light absorbance range or absorption coefficient, and controlling the solution properties and film formation kinetics.^10−12^ Compared with host solvents, which usually possess high solubility for both donor and acceptor molecules, additives typically have a selective solubility to one of the components and less volatility due to higher boiling points.^13^ Presently, most additives are liquids, such as for example 1.8-diiodooctane (DIO), 1-chloronaphthalene (CN), 1,4-difluorobenzene (DFB), and diphenyl ether (DPE).^14−16^ Besides liquid additives, solid additives have also attracted considerable attention owing to their various advantages, including directing the morphology, assisting a layer-by-layer processing fabrication, and requiring simple post treatments as well as improving device efficiency and stability.^17−19^

**Figure 1:**
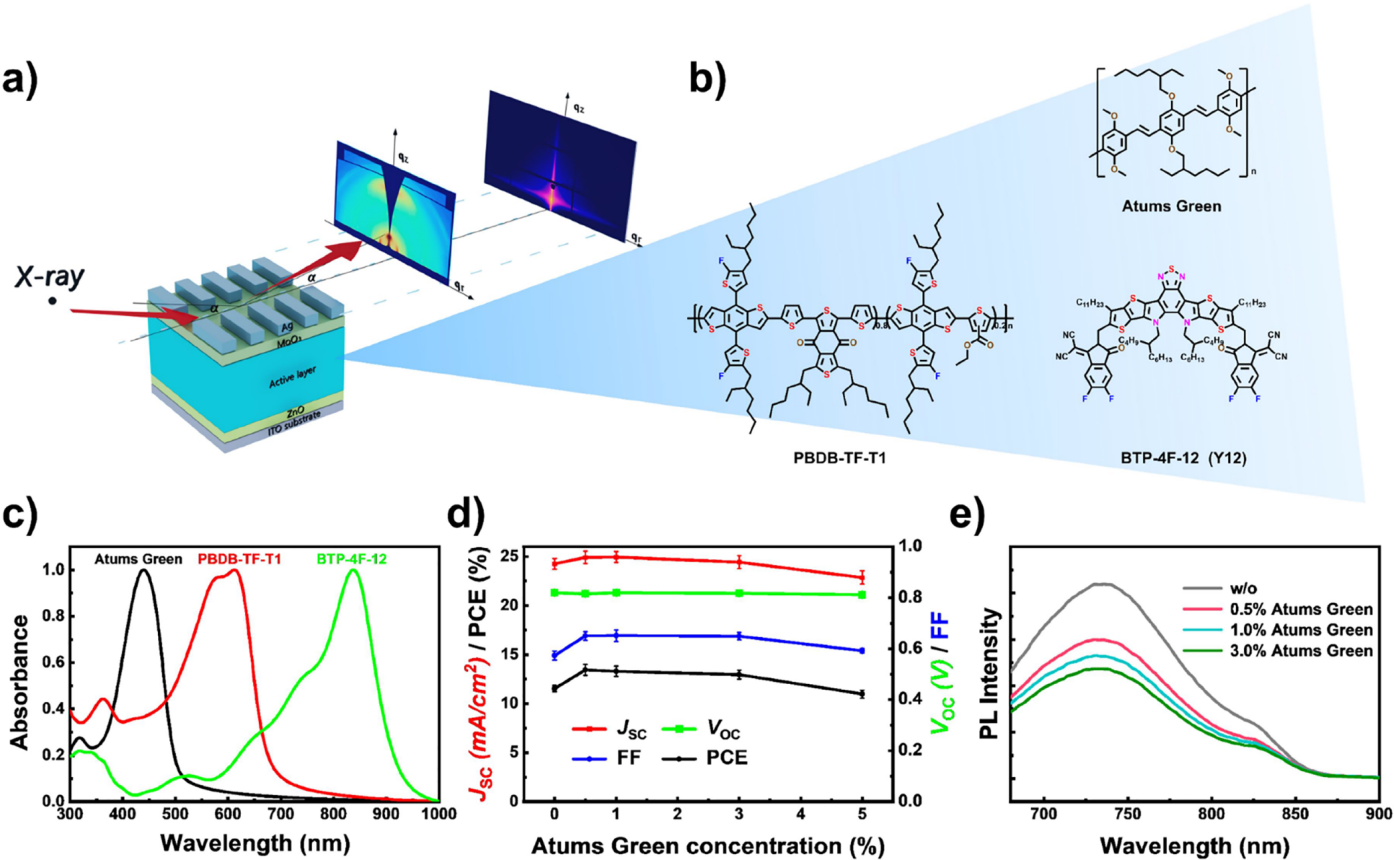
(a) Scheme of *operando* measurement setup, (b) chemical structures of materials used in this work. (c) UV–vis spectra of materials used in this work, (d) device performance of PBDB-TF-T1:BTP-4F-12 solar cells with Atums Green as additive, (e) PL spectra of PBDB-TF-T1:BTP-4F-12 films with Atums Green as additive.

Another issue calling attention to present research on organic solar cells is their environmental-friendliness.^20,21^ Most solvents applied now are halogenated (e.g., chloroform, chlorobenzene, and ortho-dichlorobenzene) or aromatic (such as 1,2,4-trimethylbenzene, *o*-xylene, and toluene). Common additives, such as DIO, CN, DFB, and DPE, are mostly halogenated or aromatic.^22−27^ The inevitable toxicity from the evaporation of the solvent and additive can harm the human body and cause pollution during device fabrication, operation as well as waste solvent disposal. Therefore, in this study, we select a solvent that is neither halogenated nor aromatic, namely tetrahydrofuran (THF). THF can be considered less environmental-unfriendly. To form the pn-junction in the active layer, the conjugated donor polymer PBDB-TF-T1 and the small molecule acceptor named BTP-4F-12 are chosen (full names in SI, see Figure 1b) since they work well together in THF.^28−31^ PBDB-TF-T1 shows better applicability compared with the very commonly used polymer PBDB-TF (sometimes called PM6) due to the introduction of PTO2 with an optimized ratio of 0.8:0.2, where PTO2 offers a better solubility in THF without a loss in the device efficiency.^28,31^ BTP-4F-12 is a derivative of the heavily used nonfullerene acceptor BTP-4F (sometimes called Y6) with a longer side chain for better solubility in THF.^30,31^ Polymer additives were also reported to have great potential in organic solar cells, such as polystyrene (PS) and poly dimethyl-siloxane (PDMS). Besides classical polymer, an additive with rich oxygen, bis(3,4-dimethylobenzylideno) sorbitol (DMDBS), was also used as a solid additive.^32,32,39^ Taking into consideration common additives such as CN or DPE, a molecule with an aromatic structure and oxygen groups has the potential to act as an effective additive for organic solar cells. In addition, nonvolatile polymer additives have shown great advantages as well. Their existence in the active layer can help to improve film stability and enhance the long-term performance of the device. Here, a green-fluorescent conjugated polymer named Atums Green is explored as the solid additive, which consists of an alkoxy-substituted 1,4-bis((*E*)-styryl)benzene repeating unit.^33^ In addition, Atums Green is also halogen-free and nonvolatile, which meets our target perfectly.

Grazing-incidence wide/small-angle X-ray scattering (GIWAXS/GISAXS) is an advanced scattering method offering valuable information on the crystal structure and microstructure of thin films.^34−36^ Classical ex situ GIWAXS/GISAXS measurements provide limited information about kinetic processes in thin films.^37−40^ In addition, so far in situ GIWAXS/GISAXS measurements are mainly focused on the film formation process rather than the aging process due to the experimental complexity. Here, *operando* GIWAXS/GISAXS measurements are carried out in this study to investigate the degradation process of PBDB-TF-T1:BTP-4F-12 solar cells during the device operation. *Operando* GIWAXS/GISAXS measurements provide valuable insights about the effect of Atums Green on the device stability.

## Results and Discussion

The scheme of the used *operando* setup is depicted in Figure 1a, where GIWAXS/GISAXS measurements are performed simultaneously with *J–V* measurements. An inverted device structure consisting of ITO/ZnO/PBDB-TF-T1:BTP-4F-12/MoO_3_/Ag is selected for the solar cells. Figure 1b illustrates the chemical structures of the materials used in this study. The wide bandgap of Atums Green of 2.645 eV (see Figure S1) suggests that it acts as an insulator additive rather than a ternary donor/acceptor in the active layer. Figure 1c shows the UV–vis spectra of the materials used in the active layer, where PBDB-TF-T1 shows two peaks located at 582 nm (0–1) and 611 nm (0–0) and BTP-4F-12 has one peak at 831 nm, which is in good agreement with the literature. Atums Green has a pronounced absorption peak at 441 nm in film, which is a bit blue-shifted compared with that in solution (487 nm as reported).^31^

Atums Green is used as an additive with concentrations from 0.5 to 5%, resulting in device performance of the solar cells comparable to the literature.^28,31,41^ Interestingly, for Atums Green concentrations up to 3%, the device performance is increased compared with the reference solar cells without additives (Figure 1d and Table S1). The Atums Green doped solar cells show almost no difference in the open-circuit voltage *V*_OC_ (0.82 V) compared with the reference cells regardless of the concentration, suggesting that its addition does not affect the energy losses from the difference of the energy levels. The short-circuit current *J*_SC_ shows a slight increase around 0.7 mA/cm^2^ for 0.5 and 1.0% Atums Green doping, which might come from the tiny expansion of the absorption range from Atums Green or an improved film morphology. The improved power conversion efficiency PCE is mainly attributed to the increased fill factor FF from 0.57 to 0.65 with Atums Green concentrations from 0.5 to 3.0%, which might result from an optimized film morphology that will be discussed later. As a result, devices with 0.5% Atums Green as an additive have an improved PCE of 13.45% compared to 11.54% for the reference. Such an optimized PCE is also comparable to that of the classical DPE doped case, which is reported in our recent work.^41^ Interestingly, when further increasing the concentration of Atums Green to 5%, all three device parameters *V*_OC_, *J*_SC_, and FF, decrease sharply, resulting in a lower efficiency of 10.98%. Such a sharp decrease may be due to the large amount of ether groups in the structure of Atums Green, which may produce radicals under light irradiation at a high concentration and cause unbalanced microstructure of the active layer.^10,42^Figure 1e shows the photoluminescence (PL) spectra of PBDB-TF-T1:BTP-4F-12 films with different Atums Green doping concentrations. The decreased intensity of the PL spectra in the case of Atum Green doping suggests a promoted charge transfer between donor and acceptor, contributing to increased device performance which is observed at lower concentrations of the additive.^43,44^

**Figure 2:**
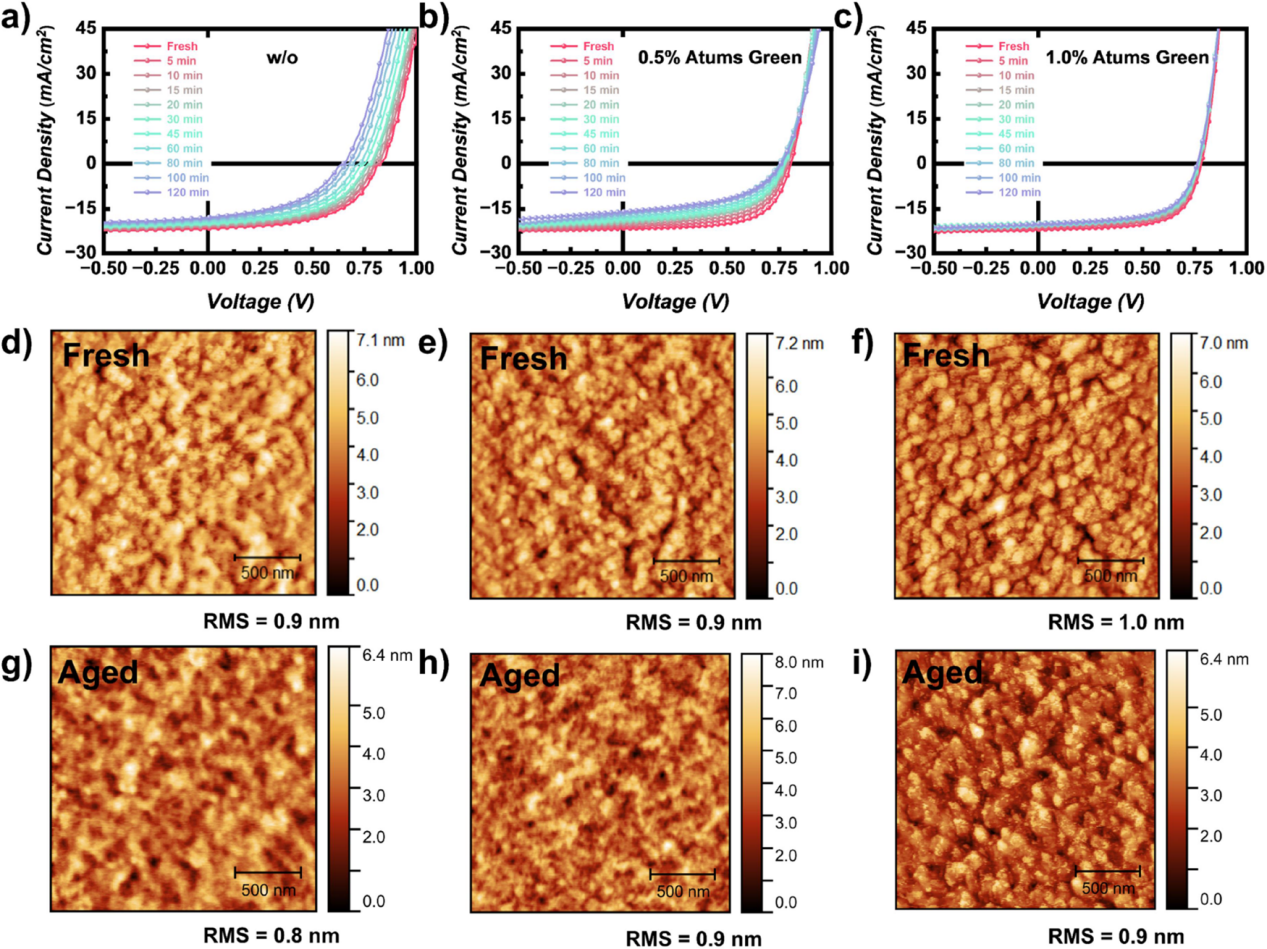
*J–V* curves of (a) PBDB-TF-T1:BTP-4F-12 solar cells without any additive, (b) PBDB-TF-T1:BTP-4F-12 solar cells with 0.5% Atums Green, and (c) PBDB-TF-T1:BTP-4F-12 solar cells with 1.0% Atums Green. AFM images of (d) and (g) fresh/aged PBDB-TF-T1:BTP-4F-12 films without any additive, (e) and (h) fresh/aged PBDB-TF-T1:BTP-4F-12 films with 0.5% Atums Green as additive, and (f) and (i) fresh/aged PBDB-TF-T1:BTP-4F-12 films with 1.0% Atums Green as additive.

Concerning real-world applications beyond device efficiency, device stability is of particular importance, where the active layer plays an essential role.^45,46^ Accordingly, we compare the stability of the solar cells with different Atums Green doping of 0.5 and 1% with the undoped reference. The evolution of the *J–V* curves is shown in Figure 2a*–*c, and the evolution of the solar cell parameters (*V*_OC_, *J*_SC_, FF, PCE) for these cells during aging is provided in Figure S2. Due to the applied conditions (in air), the reference solar cell decays strongly during aging. The *J*_SC_, *V*_OC_, and FF all decrease to ∼80% of their original values, resulting in a decrease of PCE by 50% after 2 h of aging. The devices with a tiny amount of Atums Green doping show a significantly improved stability compared with the reference case. 0.5% Atums Green suppresses the fast decay of the *V*_OC_, which remains at 93% of the initial value after 2 h of aging, while the *J*_SC_ and FF still show a similar decay trend to 80% so that the PCE decays to 58% of its initial value. In comparison, 1.0% Atums Green doped solar cells show a further improved stability, where the *J*_SC_, *V*_OC_, and FF decrease to only 97, 92, and 92% of their initial values, respectively. Thus, the overall PCE retains 83% of the start value. The decay of the *V*_OC_ might arise from the recombination of charge carriers or a decreased built-in potential.^47−49^ The loss of the *J*_SC_ might be due to the increase of domain sizes and distances in the internal active layer morphology during aging.^39,40^ The fast decay of the FF might be related to the evolution of small domains where charge carrier recombination acccumulates.^39,40^ Accordingly, the changes can be caused by a transformation of the active layer morphology and crystal structure of the donor and acceptor domains. In addition, the solar cells with Atums Green as additives show a similar degradation trend compared with using DPE as an additive in our earlier work.^41^ The similar doping effect of Atums Green and DPE demonstrates its great potential.

The surface topography before and after aging is observed with atomic force microscopy (AFM), as shown in Figure 2d*–*i. The roughness does not change much with the addition of Atums Green and after aging. Almost no difference is found for the films with Atums Green as well as for the aged ones. However, the reference case shows a more severe change after aging compared with the samples with Atums Green, which explains well its better stability. The improved phase separation with Atums Green doping and the enhanced stability after aging, as seen in the topography, indicate the observed improvements in the device performance and stability. UV–vis spectra of donor or acceptor mixed with 1% Atums Green are shown in Figure S3, where the films with Atums Green show a lower 0–1/0–0 intensity ratio for both donor and acceptor, suggesting the increased J-type aggregation with the introduction of Atums Green, which is favorable for the device performance. Furthermore, the effect of Atums Green on the donor PBDB-TF-T1 is more significant compared with the acceptor, illustrating that the additive Atums Green tends to interact with the donor more than with the acceptor for fresh solar cells. During aging, UV–vis spectra are also measured for the reference sample and 1.0% Atums Green doped films as shown in Figure S4. The Atums Green doped case also shows a lower 0–1 peak compared with the reference one. During aging, for both samples, the spectral changes are minor and very similar: The 0–1 peak of the polymer donor disappears gradually with aging time, and the intensity of the acceptor signal increases slightly in the range from 650 to 750 nm. Therefore, we conclude that the degradation does not arise from a chemical reaction of the organic active layer components, which otherwise would cause strong changes in the absorbance. Rather, the observed spectral changes indicate the rearrangement of the aggregation state in the active layer during aging. From previous studies, the interfacial degradation from ZnO was found to be one key reason for light degradation, where UV–vis spectra showed a very obvious decrease during aging.^50−53^ While such an interfacial degradation was found mainly within several nanometers, here we can further conclude that the change in the active layer is not from the chemical reaction but from the morphology change, i.e., such an interfacial degradation does not spread too deeply into bulk. For a detailed analysis of the change in the active layer, we use *operando* GIWAXS and GISAXS measurements to reveal crystal structure and microstructure changes during aging.

**Figure 3:**
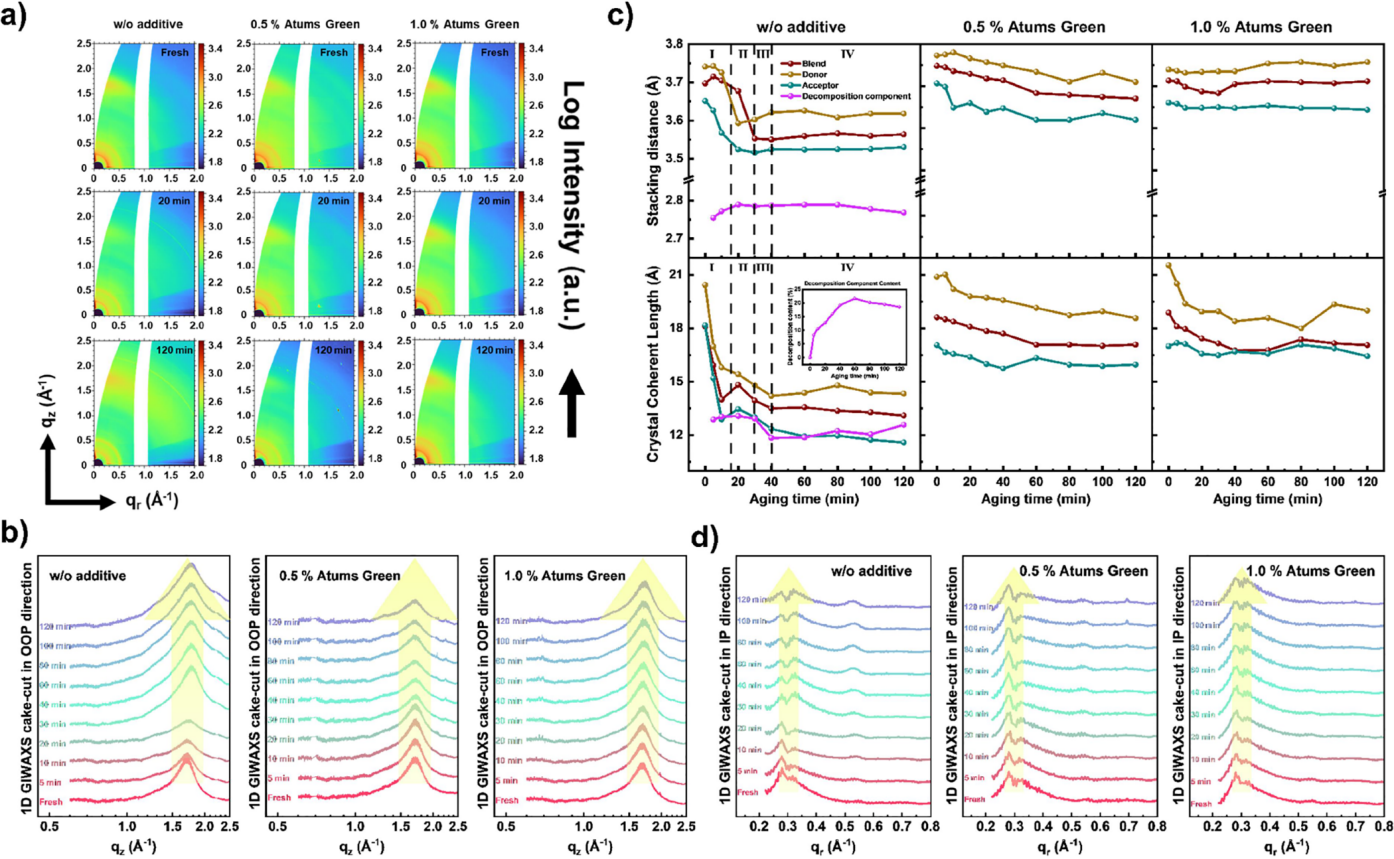
(a) Selected *operando* 2D GIWAXS data for fresh, 20 and 120 min aged solar cells based on PBDB-TF-T1:BTP-4F-12 active layers with Atums Green as additive; (b) 1D cake cuts in the OOP direction from the *operando* 2D GIWAXS data; (c) temporal evolution of lattice spacing *d* and crystal coherence length *L*_C_ of the blend, donor, acceptor, and a decomposition component during aging; (d) 1D cake cuts in the IP direction from the *operando* 2D GIWAXS data. The 1D cake cuts are shifted along the *y*-axis for clarity of the presentation.

Driven by the superior device performance, we choose 0.5 and 1.0% Atums Green doped solar cells for the *operando* study and compare them with the reference solar cell. As explained in earlier work, the ZnO/ITO substrate used is measured as the background.^41^ The signals from the Kapton windows of the *operando* chamber were subtracted in later data analysis. Figure 3a shows selected 2D GIWAXS data for the fresh and aged solar cells at 0, 20, and 120 min aging. More *operando* 2D GIWAXS data are shown in Figures S5–S7 for the studied devices. The obvious signal in the out-of-plane (OOP) direction with a q position at around 1.70 Å^–1^ refers to the π–π stacking (010) with a face-on orientation, while the signal in in-plane (IP) direction refers to the lamellar stacking (100) with a face-on orientation. The face-on orientation promises a good charge transfer in the vertical direction, which is favorable for device performance enhancement in organic solar cells.^54^ Azimuthal cake cuts in the OOP direction are carried out for the 2D GIWAXS data to extract further information (Figure 3b). The lattice spacing *d* (stacking distance) is calculated as *d* = 2π/*q,* and the crystallite coherence length (*L*_C_) is calculated as *L*_C_ = 2π*k*/fwhm, where *k* is the Scherrer factor (taking 0.9 here). The fwhm is the full-width at the half-maximum of the peak.^55^ A higher *L*_C_ value refers to a higher crystallinity, forming a strong intermolecular π–π stacking, which could improve the light absorption toward higher *J*_SC_ values. In addition, the charge carrier separation and charge carrier transfer are also promoted with higher crystallinity, which results in higher FF values. A higher *L*_C_ value promotes a better device performance. When considering one blend peak, the fresh devices show a slight decrease in *d* spacing (3.73 Å to 3.71 Å) as well as increased *L*_C_ value (18.10 Å to 18.87 Å) of the π–π stacking with Atums Green doping, suggesting the more compacted π–π stacking and increased crystallinity of the active layer, which is favorable for the charge transfer in vertical direction and thus improves the device performance, especially the FF. In general, the degradation process of the reference solar cell becomes apparent after 20 min of aging, where the shape and intensity of π–π stacking (010) peak both undergo a pronounced alteration. As a result, the *d* spacing of the π–π stacking (010) decreases from 3.73 to3.56 Å, and *L*_C_ decreases from 18.10 to13.10 Å sharply. In contrast, the π–π stacking (010) peak shape remains more consistent during the aging of the Atums Green doped devices. The calculated results are demonstrated in Figure 3c and Tables S2–S4, and the detailed degradation process is discussed in the following.

To explain the degradation mechanisms in detail, further analysis is performed where the π–π stacking peaks are deconvoluted into several peaks, and the corresponding details of the peak fits are shown in Figures S8–S10. Neat films of PBDB-TF-T1 and BTP-4F-12 were measured beforehand, where the peak located at 1.68 Å^–1^ was attributed to the polymer donor (*d* spacing = 3.74 Å) and the peak at 1.73 Å^–1^ to the acceptor (*d* spacing = 3.63 Å), which is also in agreement with the literature.^41,56^ Three to four Gaussian peaks (depending on the peak shapes) are used to model the measured scattering peaks, where the constant peak at a *q* position of around 1.36 Å^–1^ might come from the amorphous region of the acceptor and thus is not related to the π–π stacking that we are interested in.^57,58^ The evolution of lattice spacing *d* and of *L*_C_ is plotted in Figure 3c and the detailed values are listed in Tables S2–S4 as well. The S_π–π.D_ and S_π–π.A_ are the contents of donor and acceptor contributing to the π–π stacking, as determined in the fits of the 1D cake cuts. Their ratio is constantly around 1.2, which is close to the weight ratio as well, suggesting the credibility of the fit results. For the fresh solar cells, the *d* spacing values of the polymer donor remain the same as in the neat film, while that of the acceptor increases slightly to 3.67 Å. The closer *d* spacing values of the donor and acceptor suggest the interaction to form the bulk heterojunction (BHJ). The *d* spacings of the donor and acceptor show little differences for the Atums Green doped solar cells but the *L*_C_ value of the donor exhibits an obvious increase. At the same time, it decreases a bit for the acceptor, suggesting that the doping with Atums Green increases the crystallinity, contributing to the blend films.

The detailed degradation process can be divided into four stages for the reference solar cell. In stage I, the general *d* spacing shows almost no change, while the *L*_C_ value decreases quickly. To be specific, the *d* spacing of the donor remains stable while that of the acceptor decreases sharply. The *L*_C_ values decrease for both donor and acceptor contributions. The differences between the donor and acceptor components also agree with our previous results that the acceptor is more crucial for the solar cell decay for such an inverted organic solar cell.^41^ Simultaneously, a new component appears, and its content increases quickly, with slightly increased *d* spacing and constant *L*_C_ value. Such a new component might come from the broken pieces of active layer materials, which might come from the interfacial degradation as we found before and will act as charge trapping sites to increase the recombination in the BHJ, which would cause the loss of the *V*_OC_.^41,51^ In stage II, the general *L*_C_ value remains relatively stable while the *d* spacing undergoes a sharp decrease. The *d* spacing decreases sharply for the donor and decreases slowly for the acceptor. The evolution of *L*_C_ becomes slow for both components. The content of the decomposition component is still increasing in this stage while the *d* spacing and *L*_C_ value remain stable. Finally, in stage III, the degradation slows down where the *d* spacing and *L*_C_ values remain unchanged for both donor and acceptor. As for the decomposition component, the *d* spacing also remains stable while the *L*_C_ value shows a sharp decrease with a constant stage to follow in the next stage (stage IV). When 0.5% Atums Green is added in the active layer, the *d* spacing and *L*_C_ values become pretty stable for the acceptor but still decrease for the donor. However, the decrease is less pronounced and has a slower rate compared with the reference case. Furthermore, with 1.0% Atums Green doping, the stability has increased even more. Only the *L*_C_ value of the donor undergoes a slight decrease, where such decreased crystallinity of the polymer donor can cause the observed slight loss in the *J*_SC_ and FF values for the solar cell. Thus, it can be seen that the Atums Green addition shows a preferable acceptor selectivity during aging, of which the evolution of *d* spacing, as well as *L*_C_ values, are quickly suppressed with already 0.5% Atums Green doping.

In addition to the OOP direction, azimuthal cake cuts at the IP direction are performed as well with the background subtracted, which includes the information about the lamellar stacking of face-on oriented crystallites (see Figures 3d). Two peaks at positions *q* = 0.28 Å^–1^ and *q* = 0.32 Å^–1^ are attributed to the acceptor and donor with stacking distances of 22.44 and 19.63 Å, respectively, where the peak positions match with the literature.^59^ For the reference case, the difference between the two peaks becomes indiscernible during aging, and the relative intensity of donor/acceptor signals shows an obvious increase, where a sharp evolution is found after 20 min in the second stage as well. Such an evolution of the lamellar stacking might come from a split of the donor/acceptor phases or the decomposition of the acceptor, thereby lowering its signal intensity in the IP direction. In contrast, the lamellar stacking remains stable for the 0.5% Atums Green doped case, where the peaks only show a decrease in the intensity, which is referred to as the first stage of degradation as described above, suggesting that 0.5% of Atums Green slows down the degradation process by retarding the crystal structure changes. Furthermore, the 1.0% Atums Green case shows a more stable lamellar stacking during the aging, where only a little difference is found during the aging. The results further indicate the enhanced film stability caused by the Atums Green doping.

**Figure 4:**
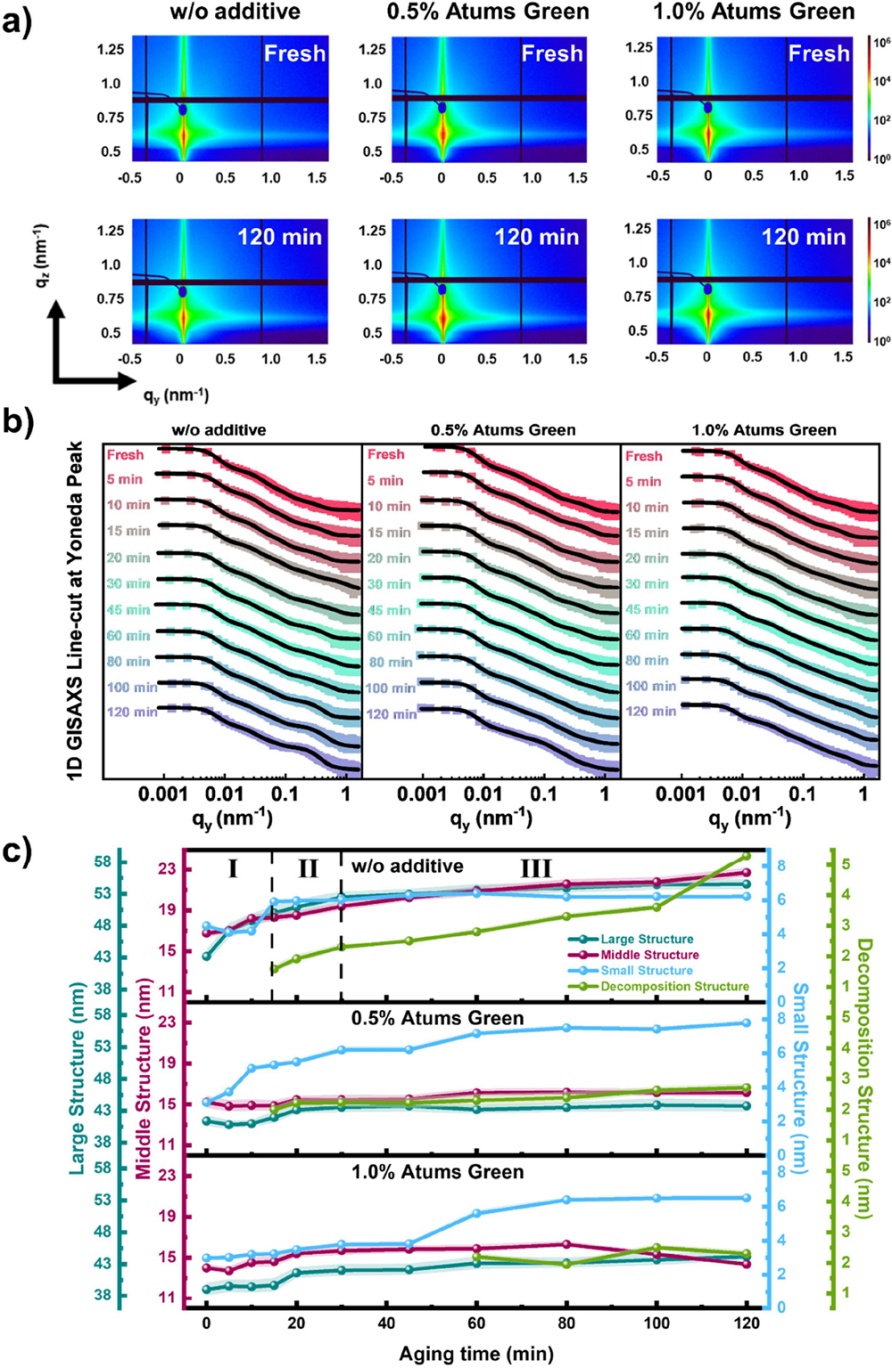
(a) Selected *operando* 2D GISAXS data for fresh and 120 min aged PBDB-TF-T1:BTP-4F-12 films with Atums Green as additive; (b) 1D horizontal line cuts at the Yoneda peak position of the *operando* 2D GISAXS data; (c) temporal evolution of characteristic structures determined in fits of the horizontal line cuts.

*Operando* GISAXS is also carried out along with the *J–V* and GIWAXS measurements to understand the changes in the active layer morphology during device operation. Selected 2D GISAXS data of fresh and aged solar cells are shown in Figure 4a. Selected other *operando* 2D data are shown in Figures S11–S13. The critical angles (α_c_) of active layer materials are calculated to be 0.135° for PBDB-TF-T1 and 0.132° for BTP-4F-12 with an X-ray wavelength of 1.044 Å, which is consistent with the calculation for PM6 and Y6 in the literature.^60^ Horizontal line cuts of the 2D GISAXS data are taken at the Yoneda peak located at 0.56 nm^–1^ (see Figure 4b, detailed analysis of the Yoneda peak can be found in our recent work).^41^ These data are modeled by assuming three to four cylindrically shaped object types (domains) in the BHJ films with different radii.^61^ The modeling is done in the framework of the distorted-wave Born approximation (DWBA) and the effective interface approximation (EIA), where a Gaussian size distribution is assumed to account for the polydispersity of the domains.^61^Figure 4c summarizes the changes of domain radii during operation for these three cells, where the middle and small domains are more crucial for the solar cell performance since their dimensions are closer to the exciton diffusion length of the materials used in the active layer.^40^ For the reference case, similar defined stages can be found as in the GIWAXS results. In the initial 15 min, defined as the first stage, all three original domains enlarge rapidly, corresponding to the first stage of GIWAXS, where the crystallinity decreases, and the acceptor stacking becomes more compact. Such evolutions might result from the regrowth of polymer domains. In the second stage, the increase rate of domain sizes slows down, and a new component is detected after a period of aging, which might be related to the component detected by analysis of GIWAXS data. This change corresponds to the sharp decreased *d* spacing and crystallinity of the donor as found by GIWAXS. Next, these three domain sizes no longer change, while the size of the decomposition component is still increasing, which causes the continuous decay of *V*_OC_. A separation of the decomposition process into the third and fourth stages, like in GIWAXS, is not evident for the material morphology probed with GISAXS. For the 0.5% Atums Green doped solar cell, the temporal evolution of the large and middle-sized domains is suppressed, while the smallest domain becomes larger during aging. Such behavior suggests that the evolution of the smallest domain is the key reason for the loss of the *J*_SC_ and FF, with the FF loss being the most detrimental for the devices. Furthermore, the decomposition component also appears at the same time as for the reference case, which could further confirm our assumption that it originates from the inevitable interfacial degradation, while its size remains constant afterward, which means that the decomposition happens to some extent but then gets stopped. Such behavior can be the reason for the observed improved stability in the *V*_OC_. For the 1.0% Atums Green doped solar cell, a similar stability is found for the two large domains as for 0.5% doping. In contrast, the smallest domain size still undergoes a slight increase, which is postponed. Since the degree of change is significantly lower than for the other two cases, the structure stability observed in the morphology appears to be the key to the stability of the *J*_SC_ and FF, with both equally contributing to the aging. The decomposition component appears later in the degradation process, which further underlines the enhanced device stability upon adding 1.0% Atums Green.

## Conclusions

Additives are important in directing the film morphology and device performance of organic solar cells, particularly solid additives, which are interesting as they remain inside the active layers. The present work explores a green-fluorescent conjugated polymer denoted Atums Green is explored as a solid additive in green-solvent based organic solar cells based on the donor:acceptor blend PBDB-TF-T1:BTP-4F-12. For solar cells, the device performance and stability are investigated. Atums Green doped solar cells show an increased device performance in terms of higher power conversion efficiencies and enlarged operational stability. A promoted charge transfer and less recombination due to the additive mainly contribute to the increased FF. The degradation during device operation does not result mainly from the chemical redox reaction, although the devices are operated in air, but from changes in the crystallinity and microstructure. During aging in air under illumination, *operando* GIWAXS and GISAXS are applied to investigate such an evolution of the active layer structure. A four-stage degradation process is found for the reference cell without additives. In stage I, the *d* spacing of the π–π stacking decreases gradually, while the *L*_C_ value decreases sharply, in conjunction with fast-increasing domain sizes, which causes the loss of *J*_SC_ and FF. Moreover, a decomposition component is also detected during the first stage, which is linked with the decrease of the *V*_OC_. In the second stage, the *L*_C_ value remains relatively stable while the *d* spacing shows a sharp decrease. In the third stage, the degradation slows down with a relatively stable *d* spacing, *L*_C_ and domain sizes until in the fourth stage where the final aged film is reached. With 0.5% Atums Green doping, the evolution of *d* spacing and *L*_C_ during the first stage is slowed down, and the decomposition component is suppressed, which stabilizes the *V*_OC_ during device operation. 1.0% Atums Green doping further stabilizes the evolution of crystallinity and suppresses the decrease of the *J*_SC_ and FF. The detailed evolution of the π–π stacking signal of donor and acceptor shows that Atums Green is more effective for the acceptor component. In summary, this study utilizes operando strategies to probe the morphological evolution during solar cell aging and correlates the morphological properties (including molecular stacking, crystallinity, molecular aggregation) to the device performance, thereby offering a deep understanding of the degradation mechanisms of organic solar cells. The solid additive Atums Green with only 1.0% enhances the device performance and operational stability, paving the way for fabricating highly efficient and stable organic solar cells, which can operate in air, demonstrating the great potential in green-solvent-based organic solar cells.

## Methods

### Materials and Device Fabrication

The patterned ITO substrates were purchased from Liaoning Youxuan New Energy Technology Co., Ltd. The substrates were ultrasonically cleaned before using in the sequence of diluted Hellmanex III (2:98), DI water, acetone, and isopropanol consecutively for 30 min each. Cleaned ITO substrates were then flow-dried and treated with an O_3_-plasma (0.4 mbar, 10 min) before spin-coating.

The ZnO nanoparticles (NPs) solution was synthesized as reported.^62^ The ZnO NPs were dispersed in methanol with 0.05% (volume fraction) ethanolamine. The concentration was 15 mg/mL. The ZnO NPs solution was spin-coated on the cleaned ITO substrates with a speed of 3000 rpm, getting an optimized thickness of around 30 to 40 nm. The as-cast ZnO films were then annealed at 130 °C for 10 min.

Poly[(2,6-(4,8-bis(5-(2-ethylhexyl-3-fluoro)thiophen-2-yl)-benzo[1,2-*b*:4,5-*b*′]dithiophene))-*alt*-(5,5-(1′,3′-di-2-thienyl)-5′,7′-bis(2-ethylhexyl)benzo[1′,2′-c:4′,5′-c′]dithiophene-4,8-dione)]-*ran*-poly[(2,6-(4,8-bis(5-(2-ethylhexyl)thiophen-2-yl)-benzo[1,2-*b*:4,5-*b*′]dithiophene))-*alt*-(2,2-ethyl-3(or4)-carboxylate-thiophene)] (PBDB-TF-T1) and 2,2′-((2*Z*,2′*Z*)-((12,13-bis(2-butyloctyl)-3,9-diundecyl-12,13-dihydro-[1,2,5]thiadiazolo[3,4-*e*]thieno[2″,3″:4′,5′]thieno[2′,3′:4,5]pyrrolo[3,2-*g*]thieno[2′,3′:4,5]thieno[3,2-*b*]indole-2,10-diyl)bis(methanylylidene))bis(5,6-difluoro-3-oxo-2,3-dihydro-1*H*-indene-2,1-diylidene))dimalononitrile (BTP-4F-12) were purchased from 1-Material Inc. Tetrahydrofuran (THF) and diphenyl ether (DPE) were bought from Sigma-Aldrich Inc.

Atums Green was synthesized following an established procedure based on a Suzuki cross-coupling polymerization reaction as described in the literature.^33^ We used a polymer with a molecular weight of *M*_n_ = 16.5 kg/mol and a PDI = 2.5.

Atums Green was dissolved in THF at first with a concentration of 9 mg/mL. The solution was stirred overnight and then added to the blend solution of PBDB-TF-T1:BTP-4F-12 (the concentration of PBDB-TF-T1 and BTP-4F-12 was 18 mg/mL in total). The blend solutions in THF were stirred for 3 h before use. The solution was dynamically spin-coated on ZnO substrates with a speed of 2000 rpm, resulting in an optimized thickness of around 100 nm. The as-cast films were then annealed at 100 °C for 10 min.

MoO_3_ and silver were bought from Carl Roth GmbH + Co. KG. MoO_3_ was thermally evaporated on the active layer with a thickness of 10 nm. Silver was then evaporated on the top with a thickness of 100 nm. Both evaporations were conducted under a vacuum of 3 × 10^–6^ mbar.

### Characterizations

Details of the *J*–*V*, *operando J–V*, photoluminescence (PL) and atomic force microscope (AFM) measurements are provided in the Supporting Information. Gwyddion was used as the software for data analysis and image postprocessing.^63^

*Operando* GIWAXS/GISAXS experiments were carried out at the PETRA III synchrotron P03 beamline at Deutsches Elektronen-Synchrotron (DESY, Hamburg).^64^ A monochromatic X-ray beam with an energy of 11.7 keV and a beam size of 23 × 32 μm^2^ was applied. The samples were probed at an incidence angle of 0.13° with a SDD of 164 m. The exposure time for each GIWAXS image was 1 s. Alignment was carried out every 15 min to precisely maintain the value of the incidence angle, which otherwise can change due to the thermal expansion of glass substrate. The positions of the X-ray beam center and sample-to-detector distance (SDD) in all measurements using a LAMBDA 9 M (X-Spectrum, pixel size 55 μm) detector were calibrated by fitting the patterns of LaB_6_ and CeO_2_ with the DPDAK package.^65^ The reshaped 2D GIWAXS patterns and the 1D cake cuts of the scattering data were processed with the Python tool INSIGHT.^66,67^ The scattering signal of ITO was obtained from the GIWAXS data measured at 0.6° (taken at each measurement point with the same position) for the correction of the SDD. The SDD values were done by calibrating the ITO peaks to *q* = 2.132 Å^–1^, which was determined before from the XRD measurement of the same batch ITO substrate.

For GISAXS, the samples were probed at an incidence angle of 0.4° with a Pilatus 2 M (Dectris, pixel size 172 μm) detector in an SDD of 4242 mm. The exposure time for each GISAXS image was 1 s. The 2D GISAXS data were generated with BornAgain (version 1.17.0).^68^ The line cuts of the 2D GISAXS data were finished with the DPDAK package and fitted in the framework of the distorted-wave Born approximation (DWBA) and the effective interface approximation (EIA) with a lab fitting tool.^69^

## Supporting Information Available

Additional characterization information and experimental details, including energy level diagram of materials, device performance degradation curves, UV–vis spectra, 2D GIWAXS data and 2D GISAXS data, device parameters, and fit results of OOP cake cuts in GIWAXS (PDF)
