# Supplementary material for: Suppressed Degradation Process of PBDB-TF-T1:BTP-4F-12-Based Organic Solar Cells with Solid Additive Atums Green
Source: ACS Appl Mater Interfaces. 2025 Jan 30;17(6):9475–84. doi: 10.1021/acsami.4c21699 (PMC11826503; doi:10.1021/acsami.4c21699)
Supplement: Supplementary file 1 — am4c21699_si_001.pdf [file am4c21699_si_001.pdf]

Supporting Information

Suppressed Degradation Process of PBDB-TF-T1:BTP-4F-12 Based Organic Solar Cells With Solid Additive Atums Green

Zerui Li[1,2], Sergei Vagin[3], Jinsheng Zhang[1], Renjun Guo[1,4], Kun Sun[1], Xiongzhuo Jiang[1], Tianfu Guan[1], Matthias Schwartzkopf[5], Bernhard Rieger[3], Chang-Qi Ma[2], Peter Müller-Buschbaum[1,*]

[1] Technical University of Munich, TUM School of Natural Sciences, Department of Physics, Chair for Functional Materials, James-Franck-Str. 1, 85748 Garching, Germany

[2] i-Lab & Printable Electronics Research Center, Suzhou Institute of Nano-Tech and Nano-Bionics, Chinese Academy of Sciences (CAS), Ruoshui Road 398, SEID, SIP, 215123, Suzhou, China

[3] Technical University of Munich, TUM School of Natural Sciences, Department of Chemistry, WACKER Chair of Macromolecular Chemistry, Lichtenbergstr. 4, 85748 Garching, Germany

[4] Karlsruhe Institute of Technology (KIT), Institute of Microstructure Technology, Herrmann-von-Helmholtz-Platz 1, 76344 Karlsruhe, Germany

[5] Deutsches Elektronen-Synchrotron DESY, Notkestr. 85, 22607 Hamburg, Germany

* corresponding author: muellerb@ph.tum.de

Characterizations

J-V measurements were carried out with a solar simulator (class ABA, Newport), where a lamp (SMR-100/XEAR2, Ushio America, Inc.) is applied as the light source, and 2611B Keithley is used as the monitor of voltage and current. The light intensity was corrected with a reference silicon solar cell (Fraunhofer ISE019-2015) and set at 100 mW/cm^2 before every measurement. The solar cell devices were measured by masking the active area with a metal mask of 0.079 cm^2.

Operando J-V measurements were carried out with a homemade system called "pocket solar", where a lamp (PE150AF Xenon Ceramic Body Parabolic Lamp) is adapted to imitate the solar spectrum. The atmosphere temperature during the measurement was controlled at 25 ^{o}C *via* a Julabo so that the effects from heat could be excluded. The light intensity was adjusted to 100 mW/cm^2 with a reference silicon solar cell (Fraunhofer ISE019-2015) before the measurements as consistent with the above. The *J-V* sweeps ran every two minutes, and the parameters (V_{OC}, J_{SC}, FF, PCE) were calculated and recorded automatically.

Atomic Force Microscope (AFM) images were obtained with an AFM instrument (Nanosurf, FlexAFM, Switzerland). Gwyddion was used as the software for data analysis and image post-processing.

Photoluminescence (PL) spectra were collected by PerkinElmer LS 55 Fluorescence Spectrometer under a 580 nm excitation laser.

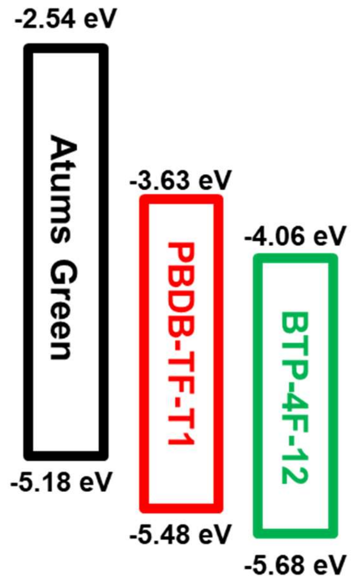

Figure S1. Energy levels of materials used in this work.

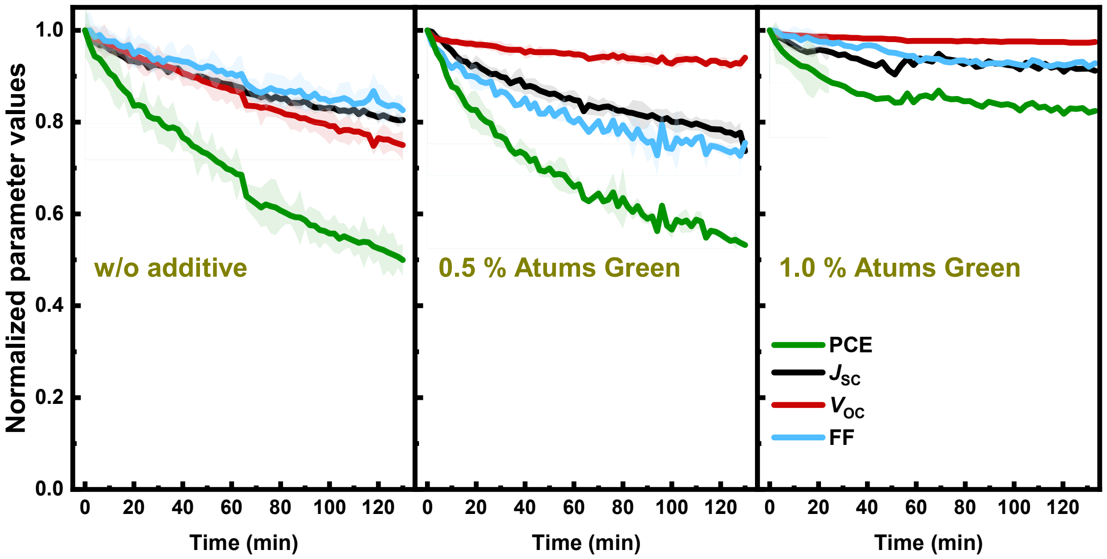

Figure S2. Temporal evolution of PBDB-TF-T1:BTP-4F-12 solar cell parameters with Atums Green as additive extracted from *JV*-curves under illumination in air (25-27 °C, 28-32% R.H.).

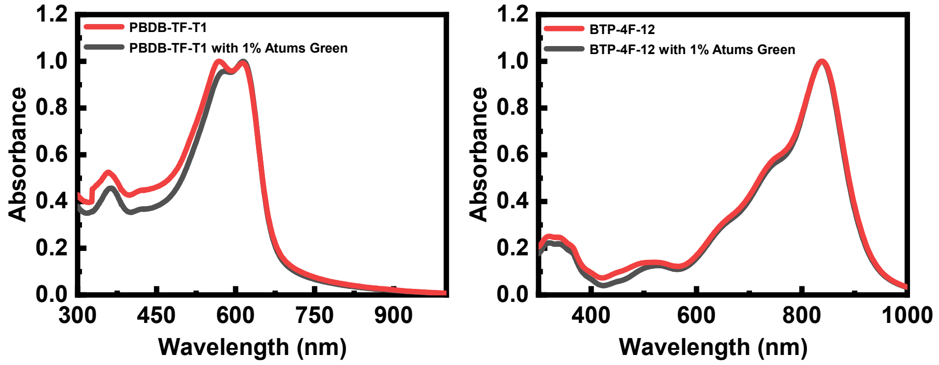

Figure S3. UV-Vis spectra of PBDB-TF-T1 (left) and BTP-4F-12 (right) films with/without 1% Atums Green additive (black/red curves).

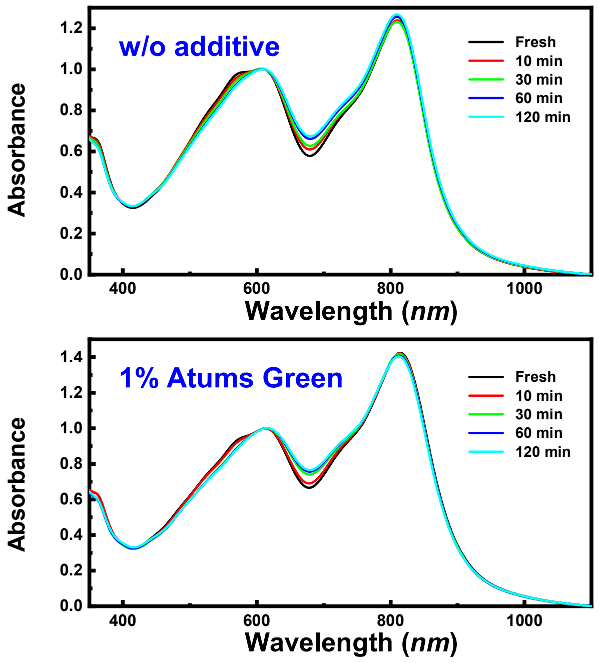

Figure S4. Evolution of UV-Vis spectra of reference (top) and 1 % Atums Green doped (bottom) PBDB-TF-T1:BTP-4F-12 films during aging.

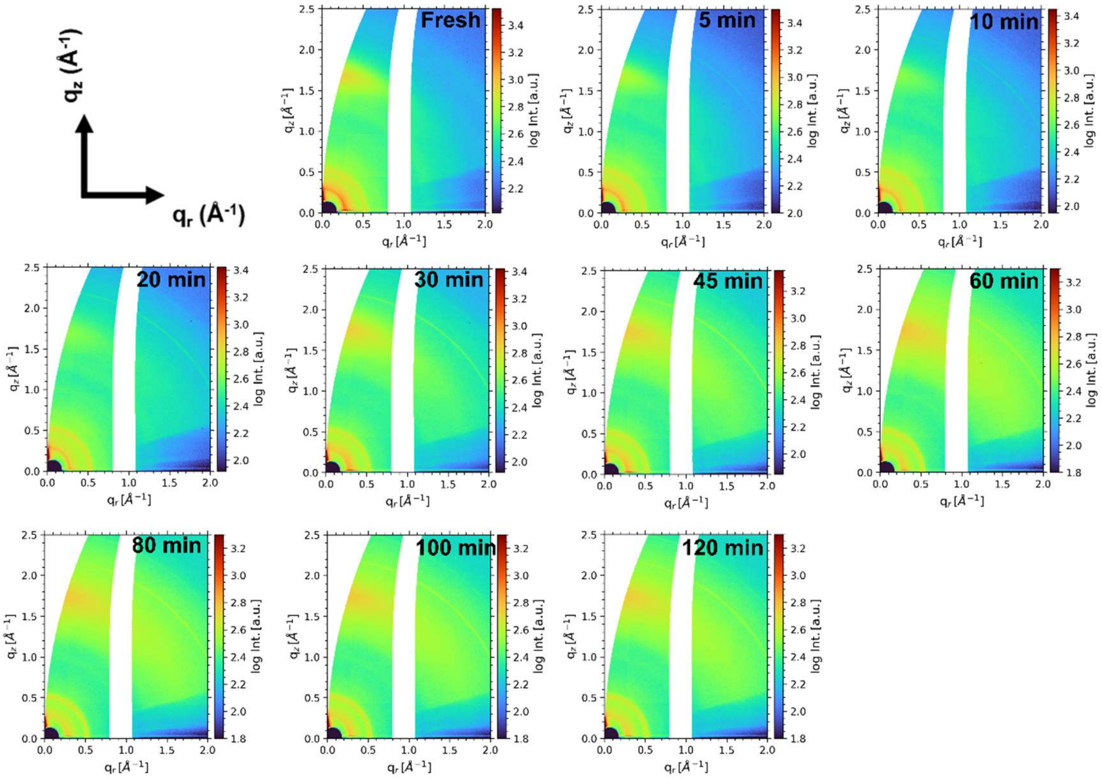

Figure S5. *Operando* 2D GIWAXS data of the reference solar cell at selected times of the device operation in air under illumination for times as indicated.

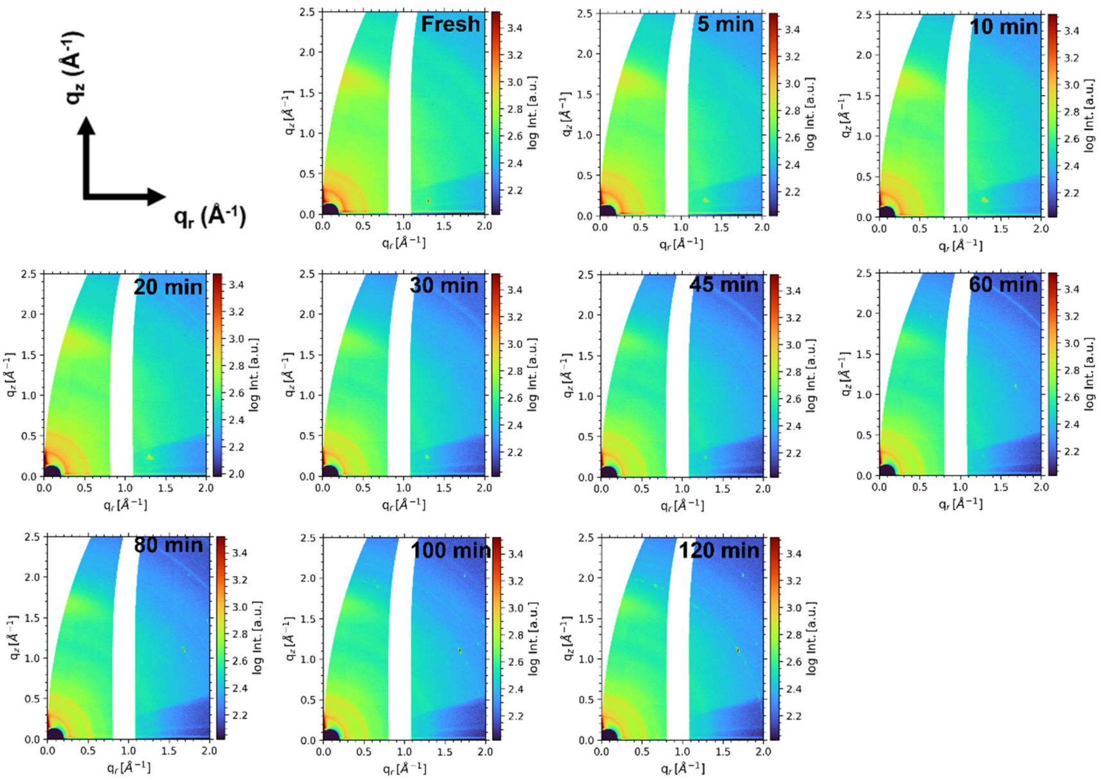

Figure S6. *Operando* 2D GIWAXS data of the 0.5% Atums Green doped solar cell at selected times of the device operation in air under illumination for times as indicated.

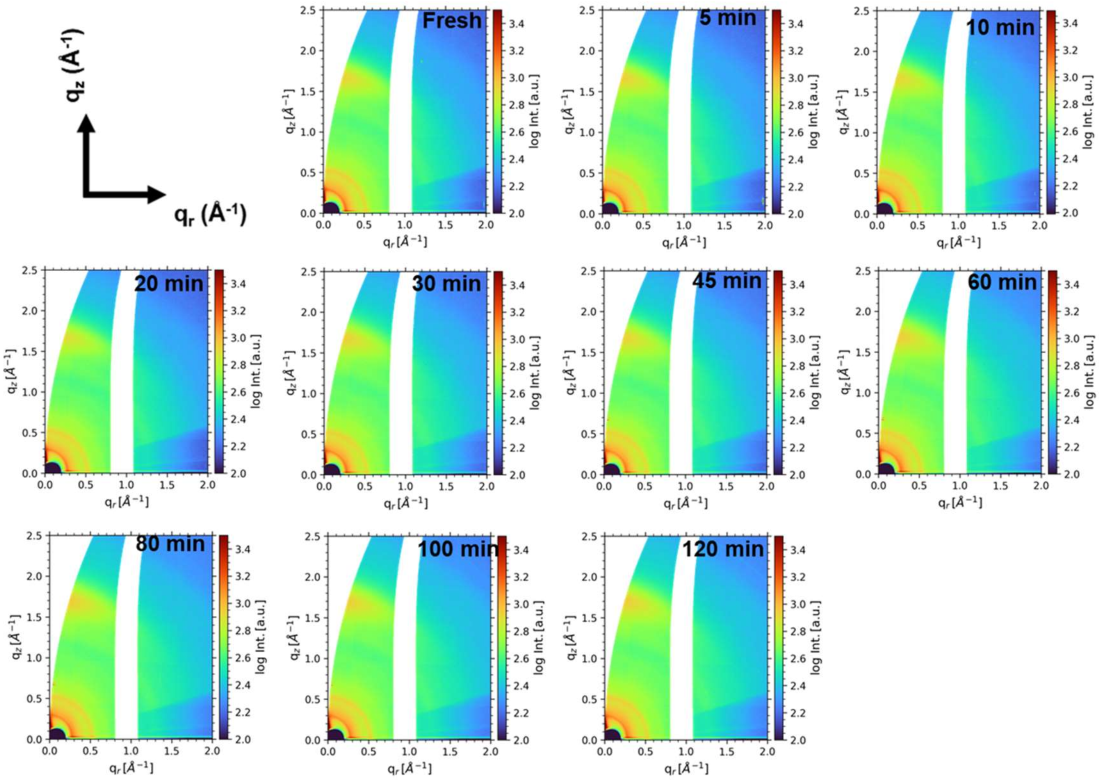

Figure S7. *Operando* 2D GIWAXS data of the 1.0% Atums Green doped solar cell at selected times of the device operation in air under illumination for times as indicated.

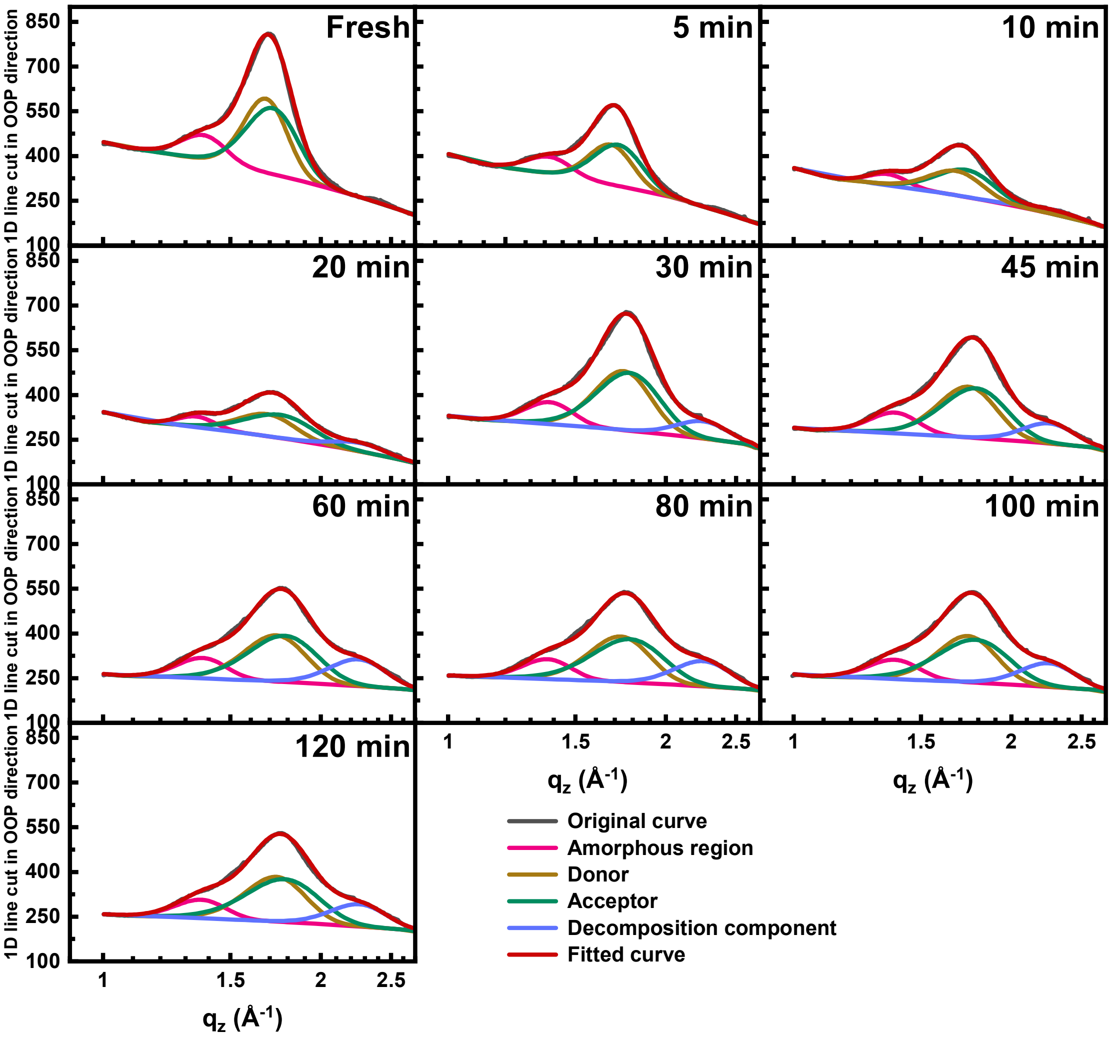

Figure S8. Fits of the π-π stacking (010) peak probed in the *operando* GIWAXS measurements of the reference solar cell.

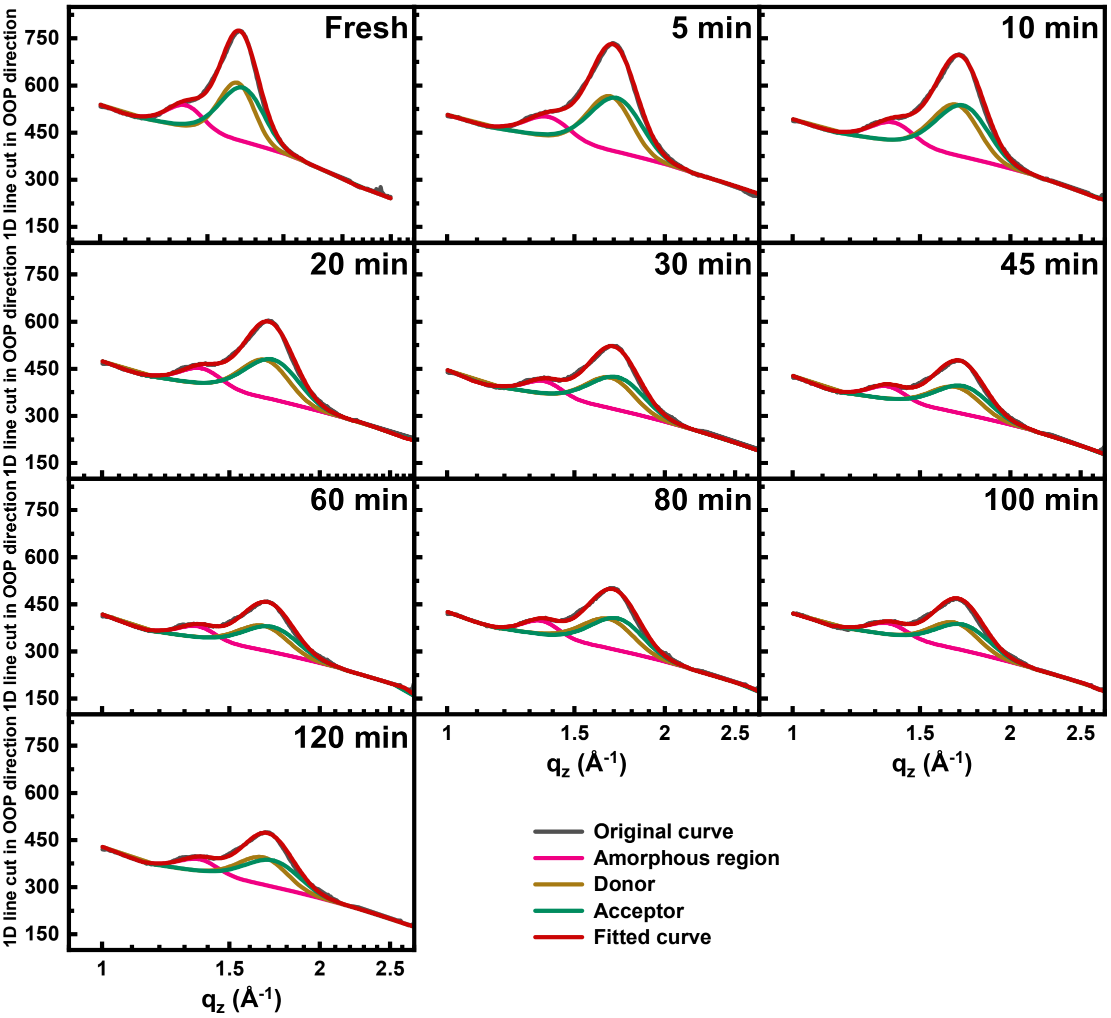

Figure S9. Fits of the π-π stacking (010) peak probed in the *operando* GIWAXS measurements of the 0.5% Atums Green doped solar cell.

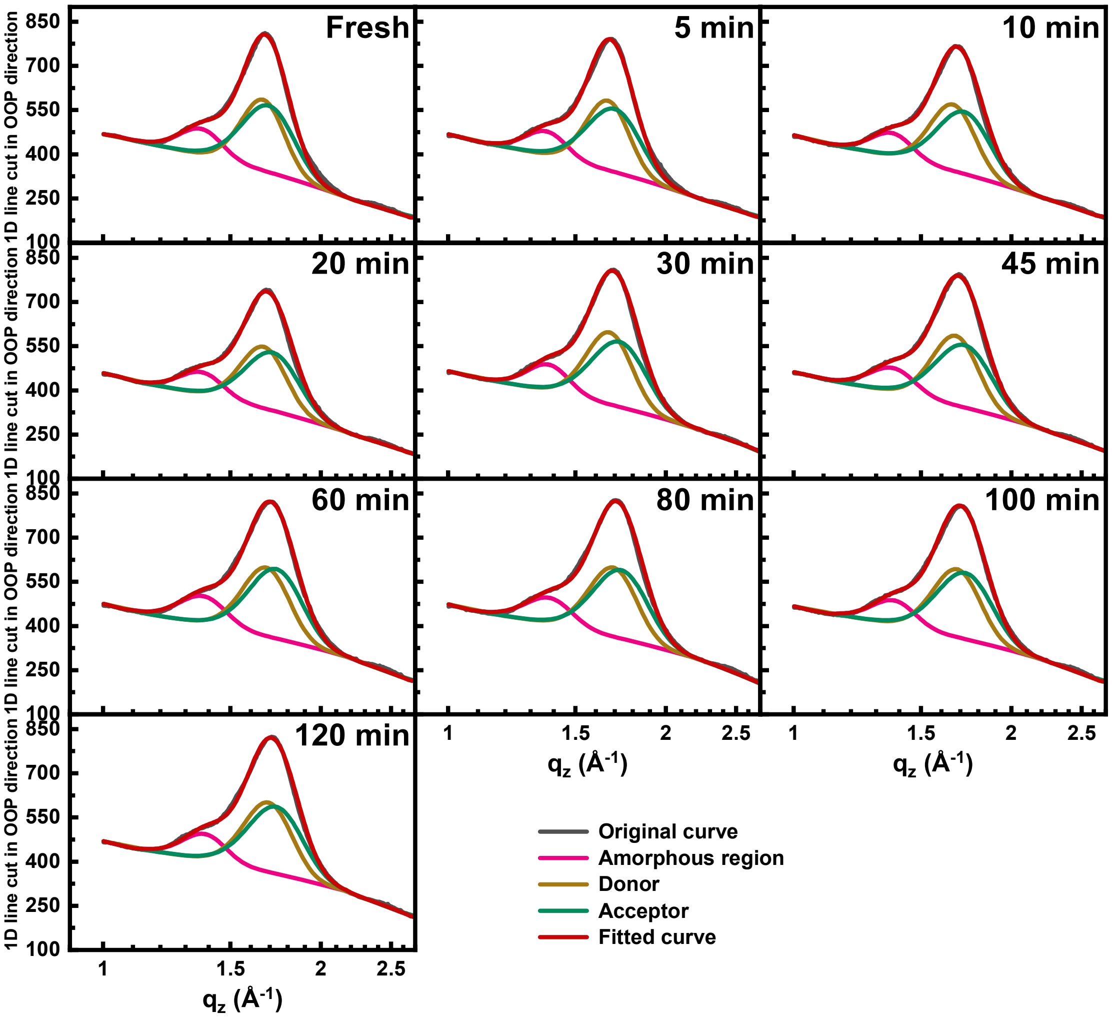

Figure S10. Fits of the π-π stacking (010) peak probed in the *operando* GIWAXS measurements of the 1.0% Atums Green doped solar cell.

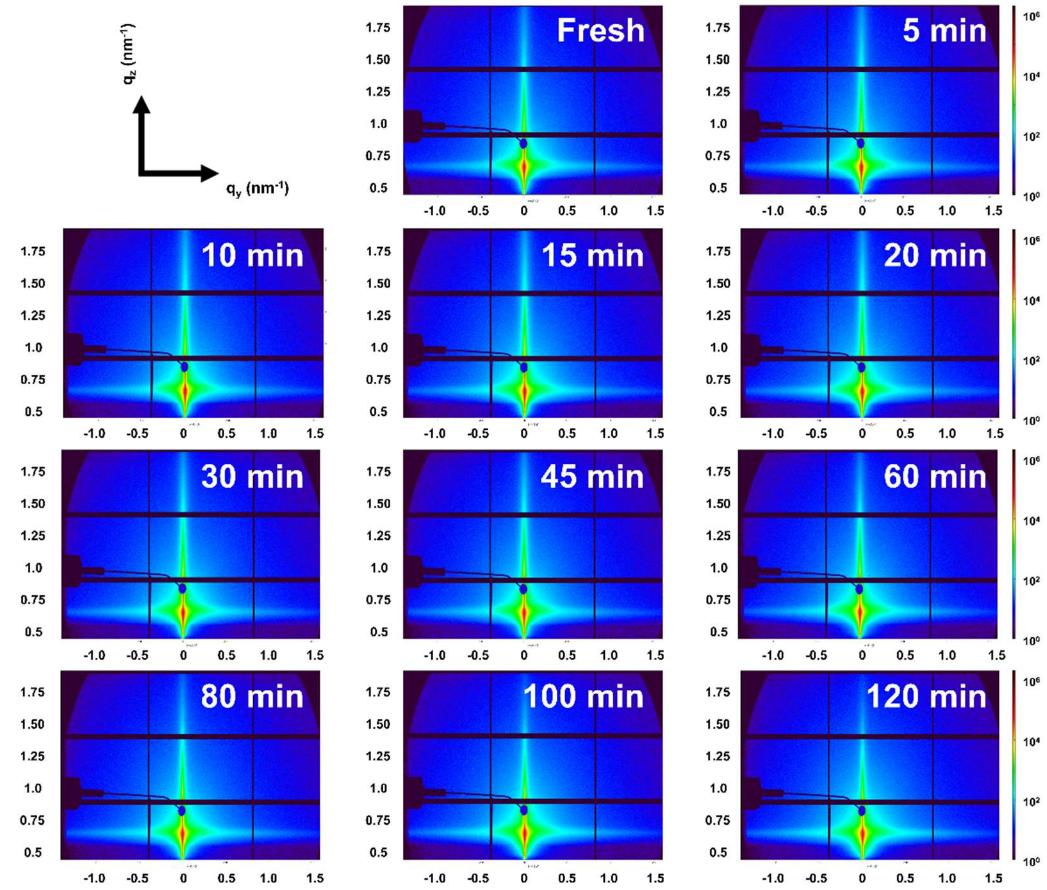

Figure S11. *Operando* 2D GISAXS data of the reference solar cell at selected times of the device operation in air under illumination for times as indicated.

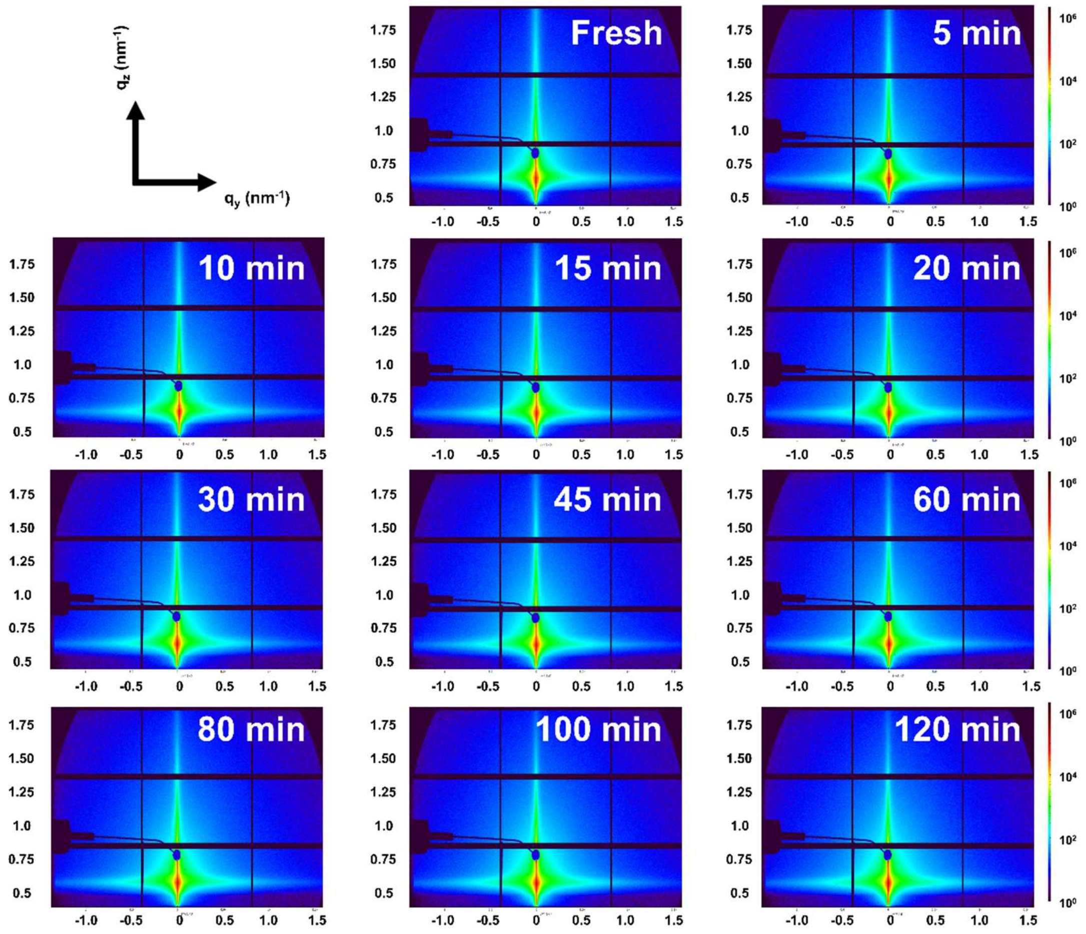

Figure S12. *Operando* 2D GIWAXS data of the 0.5% Atums Green doped solar cell at selected times of the device operation in air under illumination for times as indicated.

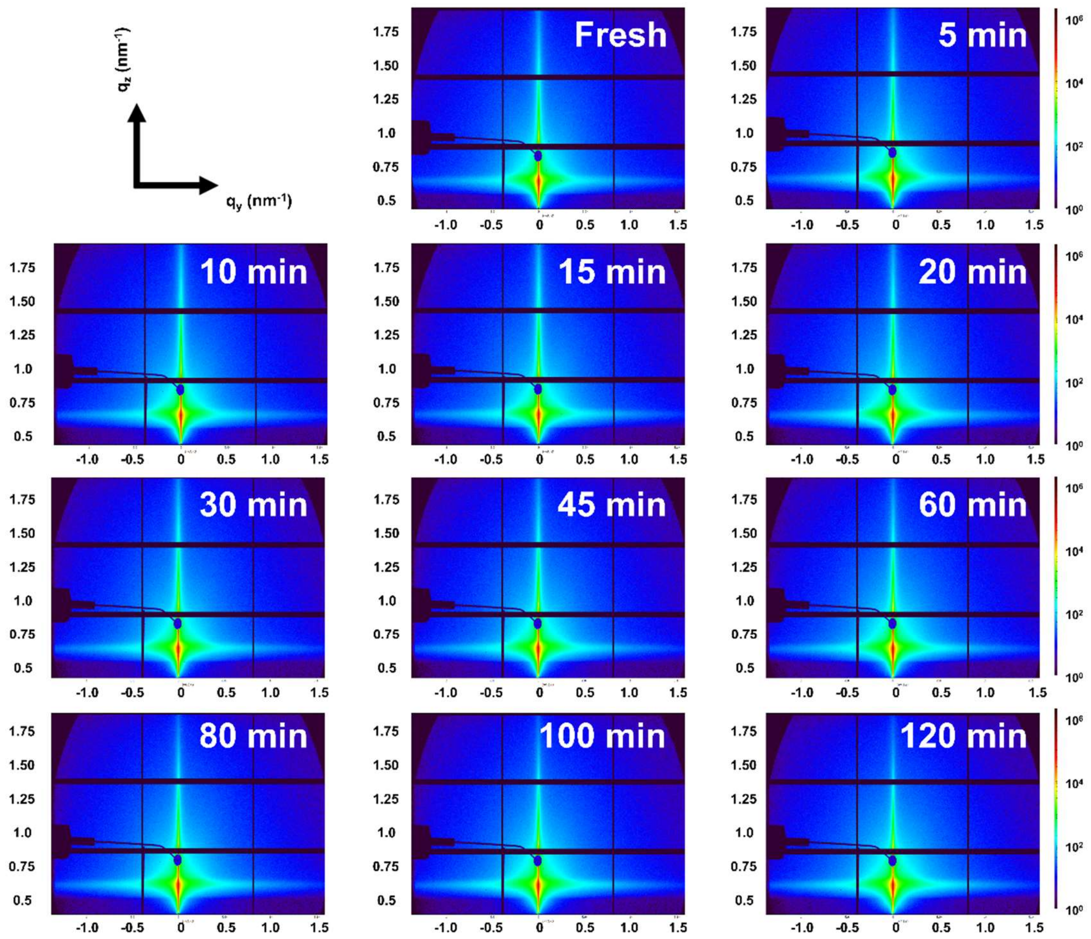

Figure S13. *Operando* 2D GIWAXS data of the 1.0% Atums Green doped solar cell at selected times of the device operation in air under illumination for times as indicated.

Table S1. Device performance of PBDB-TF-T1:BTP-4F-12 solar cells with different concentrations of Atums Green as additive

	V_{OC} (V)	J_{SC} (mA/cm^2)	FF	PCE (%)
w/o	0.819 ± 0.002	24.26 ± 0.54	0.573 ± 0.018	11.528 ± 0.316
0.5% Atums Green	0.819 ± 0.003	24.91 ± 0.63	0.655 ± 0.017	13.449 ± 0.538
1.0% Atums Green	0.819 ± 0.002	24.95 ± 0.55	0.649 ± 0.022	13.308 ± 0.514
3.0% Atums Green	0.819 ± 0.001	24.43 ± 0.67	0.645 ± 0.014	12.952 ± 0.455
5.0% Atums Green	0.812 ± 0.003	22.85 ± 0.68	0.592 ± 0.009	10.980 ± 0.414

The average PCE values with standard deviations are calculated from at least 15 devices.

All devices are tested with a metal mask applied (effective area of 0.079 cm^2)

Table S2. The evolution of the parameters fitted from out-of-plane cut in GIWAXS patterns for reference cell

**Aging time (min)**	**Blend**			**Donor**			**Acceptor**				**New component**		
	$S_{\pi\text{-}\pi.B}$ (%)	$CCL_{\pi\text{-}\pi}$ (Å)	$d_{\pi\text{-}\pi}$ (Å)	$S_{\pi\text{-}\pi.D}$ (%)	$CCL_{\pi\text{-}\pi}$ (Å)	$d_{\pi\text{-}\pi}$ (Å)	$S_{\pi\text{-}\pi.A}$ (%)	$CCL_{\pi\text{-}\pi}$ (Å)	$d_{\pi\text{-}\pi}$ (Å)	$S_{\pi\text{-}\pi.A}$ /$S_{\pi\text{-}\pi.D}$	$S_{\pi\text{-}\pi}$ (%)	$CCL_{\pi\text{-}\pi}$ (Å)	$d_{\pi\text{-}\pi}$ (Å)
Fresh	88.98	18.10	3.73	39.74	20.43	3.74	46.08	18.17	3.67	1.16			
5	87.82	15.92	3.72	36.61	16.97	3.74	45.39	15.19	3.63	1.24	8.29	12.87	2.75
10	90.31	14.00	3.70	35.24	15.79	3.73	42.97	12.89	3.57	1.22	15.52	13.04	2.77
20	90.88	14.83	3.68	36.86	15.41	3.59	41.30	13.46	3.52	1.12	12.70	13.08	2.79
30	90.65	13.95	3.55	35.30	14.81	3.60	39.29	13.02	3.52	1.11	16.03	12.93	2.79
45	90.97	13.51	3.55	32.01	14.20	3.62	36.77	12.35	3.52	1.15	19.17	11.84	2.79
60	91.03	13.56	3.56	32.33	14.38	3.63	37.06	11.93	3.52	1.15	21.64	11.87	2.79
80	90.54	13.37	3.57	32.78	14.80	3.61	37.59	11.97	3.52	1.15	20.15	12.24	2.79
100	91.12	13.28	3.56	32.93	14.39	3.62	38.64	11.74	3.53	1.17	19.52	12.04	2.78
120	91.08	13.10	3.56	33.71	14.34	3.62	38.77	11.59	3.53	1.15	18.58	12.59	2.77

The value of $S_{\pi\text{-}\pi, A}$/ $S_{\pi\text{-}\pi, D}$ stays around 1.2, which is close to the weight ratio of acceptor and donor. The constant ratio suggests reliability of the fits.

Table S3. The evolution of the parameters fitted from out-of-plane cut in GIWAXS patterns for 0.5% Atums Green doped cell.

**Aging time (min)**	**Blend**			**Donor**			**Acceptor**			
	$S_{\pi\text{-}\pi.B}$ (%)	$CCL_{\pi\text{-}\pi}$ (Å)	$d_{\pi\text{-}\pi}$ (Å)	$S_{\pi\text{-}\pi.D}$ (%)	$CCL_{\pi\text{-}\pi}$ (Å)	$d_{\pi\text{-}\pi}$ (Å)	$S_{\pi\text{-}\pi.A}$ (%)	$CCL_{\pi\text{-}\pi}$ (Å)	$d_{\pi\text{-}\pi}$ (Å)	$S_{\pi\text{-}\pi.A}$ /$S_{\pi\text{-}\pi.D}$
Fresh	87.91	18.62	3.75	40.87	20.89	3.77	47.03	17.05	3.71	1.15
5	88.84	18.51	3.74	41.42	21.02	3.78	47.42	16.66	3.70	1.14
10	88.91	18.37	3.74	41.56	20.20	3.78	47.36	16.56	3.65	1.14
20	88.54	18.08	3.73	41.39	19.80	3.77	47.16	16.39	3.66	1.14
30	88.51	17.85	3.72	41.86	19.72	3.76	46.65	16.00	3.64	1.11
45	88.56	17.70	3.71	41.83	19.58	3.75	46.69	15.74	3.65	1.12
60	87.16	17.07	3.68	39.86	19.15	3.73	47.29	16.34	3.62	1.19
80	88.02	17.08	3.68	40.72	18.74	3.71	47.30	15.96	3.62	1.16
100	89.21	17.01	3.67	41.26	18.95	3.73	47.95	15.88	3.64	1.16
120	88.23	17.08	3.67	41.85	18.57	3.71	46.91	15.96	3.62	1.12

The value of $S_{\pi\text{-}\pi,\,A}$/ $S_{\pi\text{-}\pi,\,D}$ stays around 1.2, which is close to the weight ratio of acceptor and donor. The constant ratio suggests reliability of the fits.

Table S4. The evolution of the parameters fitted from out-of-plane cut in GIWAXS patterns for 1.0% Atums Green doped cell.

**Aging time (min)**	**Blend**			**Donor**			**Acceptor**			
	$S_{\pi\text{-}\pi.B}$ (%)	$CCL_{\pi\text{-}\pi}$ (Å)	$d_{\pi\text{-}\pi}$ (Å)	$S_{\pi\text{-}\pi.D}$ (%)	$CCL_{\pi\text{-}\pi}$ (Å)	$d_{\pi\text{-}\pi}$ (Å)	$S_{\pi\text{-}\pi.A}$ (%)	$CCL_{\pi\text{-}\pi}$ (Å)	$d_{\pi\text{-}\pi}$ (Å)	$S_{\pi\text{-}\pi.A}$ /$S_{\pi\text{-}\pi.D}$
Fresh	87.36	18.87	3.71	39.81	21.58	3.73	47.54	17.00	3.66	1.19
5	87.81	18.10	3.71	40.11	20.51	3.73	47.79	17.18	3.66	1.19
10	88.37	17.96	3.70	41.09	19.39	3.73	47.27	17.12	3.65	1.15
20	88.24	17.43	3.69	40.06	18.93	3.73	48.17	16.57	3.65	1.20
30	89.24	17.15	3.68	40.04	18.95	3.73	49.18	16.48	3.65	1.23
45	87.94	16.76	3.71	39.81	18.40	3.73	48.13	16.70	3.65	1.21
60	88.49	16.78	3.71	41.00	18.58	3.75	47.39	16.57	3.65	1.16
80	88.75	17.37	3.71	41.43	17.98	3.75	47.32	17.07	3.65	1.14
100	88.14	17.16	3.71	41.11	19.35	3.74	47.04	16.87	3.65	1.14
120	87.71	17.06	3.71	41.47	18.99	3.75	46.22	16.44	3.64	1.11

The value of $S_{\pi\text{-}\pi,\ A}$/ $S_{\pi\text{-}\pi,\ D}$ stays around 1.2, which is close to the weight ratio of acceptor and donor. The constant ratio suggests reliability of the fits.
